# Germline *RUNX1* Intragenic Deletion: Implications for Accurate Diagnosis of FPD/AML

**DOI:** 10.1097/HS9.0000000000000203

**Published:** 2019-04-02

**Authors:** Nicolas Duployez, Jean-Edouard Martin, Sabine Khalife-Hachem, Ryane Benkhelil, Véronique Saada, Christophe Marzac, Nathalie Auger, Alice Marceau-Renaut, Rémi Favier, Paola Ballerini, Olivier Caron, André Baruchel, Stéphane de Botton, Claude Preudhomme, Jean-Baptiste Micol, Hana Raslova, Iléana Antony-Debré

**Affiliations:** 1 Laboratory of Hematology, Université Lille Nord de France, Lille, France; 2 INSERM UMR-S 1172, Lille, France; 3 Department of Hematology, Institut Gustave Roussy, Villejuif, France; 4 Laboratory of Hematology, Institut Gustave Roussy, Villejuif, France; 5 Inserm UMR1170, Institut Gustave Roussy, Université Paris Sud, Equipe Labellisée par la Ligue Nationale Contre le Cancer, Villejuif, France; 6 Biology and Pathology Department, Institut Gustave Roussy, Villejuif, France; 7 Assistance Publique—Hôpitaux de Paris, Laboratory of Hematology, Hôpital Trousseau, Paris, France; 8 Department of Medical Oncology, Institut Gustave Roussy, Villejuif, France; 9 Assistance Publique—Hôpitaux de Paris, Department of Hematology, Hôpital Robert Debré, Paris, France.

In 2016, the WHO classification added a new category entitled “myeloid neoplasms with germ line predisposition” to acute myeloid leukemia (AML) classification, reflecting the increasing importance of these malignancies. Diagnosis of such patients is of clinical relevance, as they can benefit of specific clinical care including hematopoietic stem cell transplantation in first complete remission and genetic counseling. Genetic analysis is imperative to avoid the use of hematopoietic stem cell transplantation from an asymptomatic mutation carrier family donor. Thus, genetic testing for genes implicated in hereditary myeloid malignancies has to be done, whatever the age of the patient, in case of suggestive personal and/or family history, this is facilitated by the expanded use of next-generation sequencing. Among these hereditary malignancies, the familial platelet disorder with predisposition to acute myelogenous leukemia (FPD/AML) was first described in 1999.^1^ Since then, increasing number of families has been reported in the literature. Patients harbor mild to moderate thrombocytopenia and a risk of around 35% to develop AML or myelodysplasia. This disease (OMIM 601399) is due to germline genetic anomalies affecting the transcription factor *RUNX1*. Missense, nonsense, and frameshift mutations of *RUNX1* have been reported as well as small and large deletions. At the time of AML diagnosis, the most frequent acquired genetic alteration implicates a second alteration of *RUNX1* (mutation on the second allele, trisomy 21 with duplication of the mutated/deleted allele or loss of heterozygosity),^2–3^ attesting to the importance of RUNX1 dosage in AML development.^4^

A 62-year-old male (Fig. 1, I-1) was hospitalized at Gustave Roussy Cancer Center for diagnosis of an AML in August 2009. He had a history of mild thrombocytopenia unexplored for decades. He did not present any other clinical phenotype. His 2 children (II-1 and II-2) and one of his granddaughter (III-2) presented also a thrombocytopenia (II-1: 100 G/L, II-2: 80 G/L, III-2: 115 G/L) with history of easy bruising. The patient was diagnosed with an AML-M0 with normal cytogenetic, wild type for *NPM1* and *FLT3*-ITD mutations. He was in complete remission after a 3 + 7 induction therapy (daunorubicin 60 mg/m^2^, Day 1 to Day 3 + cytarabin 200 mg/m^2^, Day 1 to Day 7) complicated with an invasive pulmonary aspergillosis, followed by 4 ambulatory postremission chemotherapy (45 mg/m^2^ daunorubicin, Day 1 + 60 mg/m^2^/12 h cytarabine as home subcutaneous infusions, Day 1 to Day 5) and a cord blood transplant. Unfortunately he relapsed and died in April 2011. The family and clinical history of the patient suggested inherited myeloid neoplasms associated with platelet disorders.

**Figure 1:**
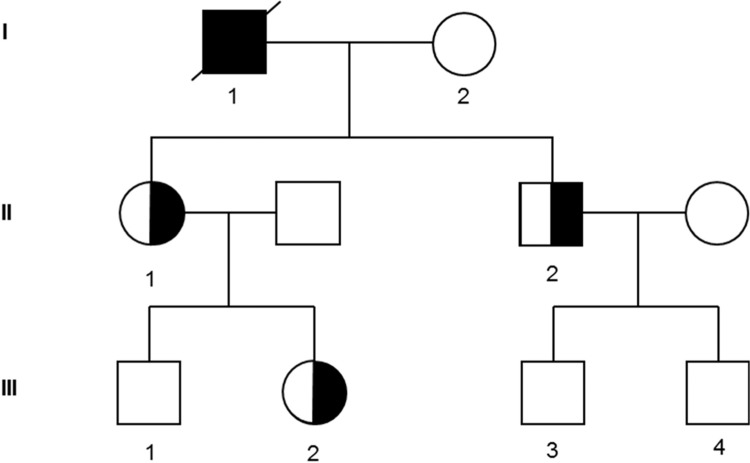
**Family tree of the affected pedigree**. The open symbols indicate pedigree members without thrombocytopenia or AML; the half black symbols, members with thrombocytopenia alone; the full black symbols, members with thrombocytopenia and AML. AML = acute myeloid leukemia.

The biological samples were collected after informed consent, in accordance with the Declaration of Helsinki. DNA was extracted from cell pellets using the QIAamp Tissue Kit (Qiagen) according to the manufacturer's instructions.

Direct sequencing of all coding exons of the *RUNX1* gene was performed as previously described by Sanger sequencing.^3^

For high-throughput sequencing (HTS), a panel of 36 genes recurrently mutated in myeloid malignancies was sequenced on genomic DNA isolated from mononuclear white blood cells. These include *ASXL1*, *BCOR*, *BCORL1*, *CALR*, *CBL*, *CSF3R*, *DNMT3A*, *ETV6*, *EZH2*, *FLT3*, *GATA2*, *IDH1*, *IDH2*, *JAK2*, *KIT*, *KRAS*, *MPL*, *NIPBL*, *NPM1*, *NRAS*, *PHF6*, *PTPN11*, *RAD21*, *RIT1*, *RUNX1*, *SETBP1*, *SF3B1*, *SMC1A*, *SMC3*, *SRSF2*, *STAG2*, *TET2*, *TP53*, *U2AF1*, *WT1*, *ZRSR2* genes. The library was prepared using the Ampliseq System according to the manufacturer's instructions and run on Ion Proton (Thermo Fisher, Waltham, MA). Raw data were analyzed with both Torrent Browser (Thermo Fisher) and SeqNext (JSI Medical System, Los Angeles, CA). A high depth of coverage (>1500) was obtained for all genes. A second panel of genes implicated in inherited myeloid malignancies including *ANKRD26*, *CEBPA*, *DDX41*, *ETV6*, *GATA2*, *RUNX1*, *SRP72*, *TERC*, *TERT*, and *TP53* was sequenced according to the same protocol.

To detect *RUNX1* deletions, SNP-array karyotyping (CytoScan HD array; Thermo Fisher Scientific) was performed according to manufacturers’ instructions. Data were analyzed with the Chromosome Analysis Suite version 3.1 (ChAS, Thermo Fisher Scientific). The intragenic *RUNX1* deletion was then confirmed by multiplex ligation probe amplification (MLPA) (SALSA MLPA P437-A1 Familial MDS-AML probemix, MRC Holland).

Based on patient's personal and familial history of thrombocytopenia, we first sequenced *RUNX1* by the Sanger method at AML diagnosis and discovered a *RUNX1* mutation (NM_001001890 exon 2 c.416G>A:p.R139Q). The R139Q mutation has been already described in FPD/AML patients.^1,5^ It affects the Runt homology domain (RHD) of RUNX1, which binds to DNA and to the other member of the CBF complex, CBFβ. After genetic counseling, we sequenced *RUNX1* in the 2 adult's relative (II-1 and II-2) presenting thrombocytopenia but unexpectedly, we were not able to find the R139Q mutation or any other *RUNX1* mutations. A clinical and biological follow-up was decided for the relatives. In 2016, we used a 36 genes-HTS panel to study the AML diagnosis sample of the proband. It confirmed the *RUNX1* R139Q mutation with a variant allele frequency (VAF) at 50%. Additional somatic mutations were described as *BCOR* p.K1137delinsTX (VAF 54%), *DNMT3A* p.W795C (VAF 48%), *NRAS* p.Q61K (VAF 47%), and *SRSF2* p.P95R (VAF 47%). Another HTS panel, including among others *ANKRD26* and *ETV6* which are also implicated in inherited myeloid malignancies with preexisting platelet disorders,^6–9^ was used but we did not find any additional mutation. Based on our recent paper describing a second aberration of *RUNX1* in AML transformation in FPD/AML patients,^2^ we hypothesized that the *RUNX1* mutation found at the AML stage was an acquired event and that we may have missed the inherited *RUNX1* alteration.

As germline *RUNX1* deletions have been already described in FPD/AML,^1,3,10–14^ we performed SNP-array and we were able to identify a microdeletion of 51 kb in the *RUNX1* gene. This result was confirmed by MLPA in the patient and in the 2 adult's relative confirming the diagnosis of FPD/AML (Fig. 2A-B). The deleted region (aminoacids 143–251) encompasses parts of the exons 3 and 4 of *RUNX1b* isoform, which encodes parts of the RHD and transactivation domains (Fig. 2C). Interestingly, a frameshift mutation in the exon 4 of *RUNX1*, resulting in a premature termination codon 22 nucleotides downstream, has already been reported in FPD/AML (*RUNX1* p.T219RfsX8) and is associated with AML development.^2,15^ This suggests that such truncated mRNA could predispose to leukemia progression in FPD/AML. Besides large *RUNX1* deletions, small deletions have been rarely described in FPD/AML, they were identified by MLPA or by CGH-array, confirming once again the importance of this type of genetic test for FPD/AML diagnosis.^11,12^ Additionally, we sequenced the proband's sample at complete remission using the 36 genes-HTS panel and did not find the mutations identified at AML stage, excepted the *DNMT3A* mutation with a lower VAF at 4%, which could represent the persistence of clonal hematopoiesis. We were not able to find the *RUNX1* mutation anymore, confirming the acquired status of this genetic event.

**Figure 2:**
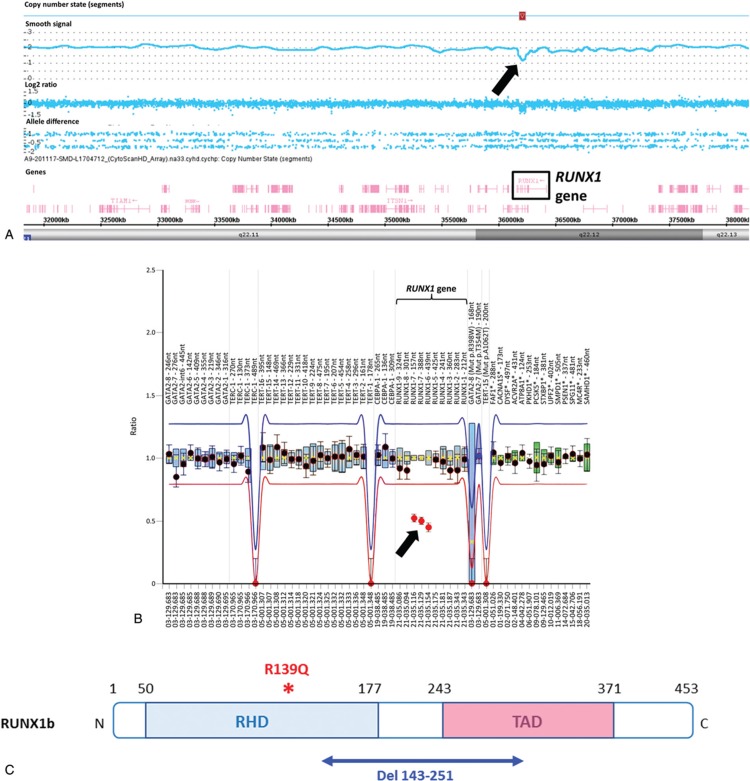
***RUNX1* alterations identified in the family**. (A) SNP-array karyotyping. View of the chromosome 21 (coordinates chr21: 31,803,844–38,217,464). The black arrow shows the deletion within the *RUNX1* gene shared by all 3 individuals (I-1, II-1, and II-2). Picture is from Chromosome Analysis suite. (B) Ratio chart of MLPA analysis. A ratio of 1 indicates no differences in respect to reference samples, while a ratio of 0.5 indicates a heterozygous deletion. Dots represent MLPA probes and lines indicate thresholds. The 3 dots, located below the lower threshold (black arrow), indicate the *RUNX1* intragenic deletion shared by all 3 individuals (I-1, II-1, and II-2). Notice that numbering of the exons 6 and 7 in the MLPA analysis corresponds to exons 3 and 4 of the *RUNX1b* isoform (NM_001001890). (C) Localization of RUNX1 abnormalities. The isoform b is represented. Germline alteration of *RUNX1* corresponding to the deletion of the aminoacids 143 to 251 (in blue) affects part of the RHD and TAD domains, whereas the acquired *RUNX1* mutation p.R139Q (in red) is located on the RHD domain, upstream of the deleted region. MLPA = multiplex ligation probe amplification, RHD = Runt homology domain, SNP = single nucleotide polymorphism, TAD = transactivation domain.

This report illustrates that, in front of a clinical and familial history evocating strongly inherited thrombocytopenia with predisposition to AML, all the biological examinations have to be conducted. Indeed, it is crucial to diagnose inherited diseases such as FPD/AML, as the management of the patient could differ from a patient with de novo AML, especially if hematopoietic stem cell transplant is considered. Moreover, it implicates a regular follow-up for patients within the family at thrombocytopenia stage, as they are at risk of developing AML. In our case, Sanger sequencing and amplicon-based HTS were not sufficient to establish the diagnosis but allowed to identify an acquired second hit affecting *RUNX1* at AML stage, confirming the association of this event with AML development. Of note, capture-based HTS may provide information on copy number variations for all homogeneously covered regions. This notion should be kept in mind in lab’ strategies especially for the assessment of haplo-insufficient genes status. In FPD/AML, as both deletions and mutations of *RUNX1* have been reported in patients, biological tests should include other techniques such as SNP-array and/or MLPA in a second phase to identify microdeletions when FPD/AML is strongly suspected. Similar strategy should be applied to diagnose other inherited hematological disorders implicating not only mutations but also gene deletions.

## References

[1] Song WJ Sullivan MG Legare RD Haploinsufficiency of CBFA2 causes familial thrombocytopenia with propensity to develop acute myelogenous leukaemia. *Nat Genet.* 1999;23:166–175. 10508512 10.1038/13793

[2] Antony-Debre I Duployez N Bucci M Somatic mutations associated with leukemic progression of familial platelet disorder with predisposition to acute myeloid leukemia. *Leukemia.* 2016;30:999–1002. 26316320 10.1038/leu.2015.236

[3] Preudhomme C Renneville A Bourdon V High frequency of RUNX1 biallelic alteration in acute myeloid leukemia secondary to familial platelet disorder. *Blood.* 2009;113:5583–5587. 19357396 10.1182/blood-2008-07-168260

[4] Antony-Debre I Manchev VT Balayn N Level of RUNX1 activity is critical for leukemic predisposition but not for thrombocytopenia. *Blood.* 2015;125:930–940. 25490895 10.1182/blood-2014-06-585513 PMC4347283

[5] Heller PG Glembotsky AC Familial platelet disorder with predisposition to acute myelogenous leukemia. *Atlas Genet Cytogenet Oncol Haematol.* 2013;17:138–143.

[6] Noetzli L Lo RW Lee-Sherick AB Germline mutations in ETV6 are associated with thrombocytopenia, red cell macrocytosis and predisposition to lymphoblastic leukemia. *Nat Genet.* 2015;47:535–538. 25807284 10.1038/ng.3253 PMC4631613

[7] Noris P Perrotta S Seri M Mutations in ANKRD26 are responsible for a frequent form of inherited thrombocytopenia: analysis of 78 patients from 21 families. *Blood.* 2011;117:6673–6680. 21467542 10.1182/blood-2011-02-336537

[8] Pippucci T Savoia A Perrotta S Mutations in the 5′ UTR of ANKRD26, the ankirin repeat domain 26 gene, cause an autosomal-dominant form of inherited thrombocytopenia, THC2. *Am J Hum Genet.* 2011;88:115–120. 21211618 10.1016/j.ajhg.2010.12.006 PMC3014357

[9] Zhang MY Churpek JE Keel SB Germline ETV6 mutations in familial thrombocytopenia and hematologic malignancy. *Nat Genet.* 2015;47:180–185. 25581430 10.1038/ng.3177 PMC4540357

[10] Beri-Dexheimer M Latger-Cannard V Philippe C Clinical phenotype of germline RUNX1 haploinsufficiency: from point mutations to large genomic deletions. *Eur J Hum Genet.* 2008;16:1014–1018. 18478040 10.1038/ejhg.2008.89

[11] Cavalcante de Andrade Silva M Krepischi ACV Kulikowski LD Deletion of RUNX1 exons 1 and 2 associated with familial platelet disorder with propensity to acute myeloid leukemia. *Cancer Genet.* 2018;222–223:32–37. 10.1016/j.cancergen.2018.01.002 29666006

[12] Jongmans MC Kuiper RP Carmichael CL Novel RUNX1 mutations in familial platelet disorder with enhanced risk for acute myeloid leukemia: clues for improved identification of the FPD/AML syndrome. *Leukemia.* 2010;24:242–246. 19946261 10.1038/leu.2009.210

[13] Sood R Kamikubo Y Liu P Role of RUNX1 in hematological malignancies. *Blood.* 2017;129:2070–2082. 28179279 10.1182/blood-2016-10-687830 PMC5391618

[14] van der Crabben S van Binsbergen E Ausems M Constitutional RUNX1 deletion presenting as non-syndromic thrombocytopenia with myelodysplasia: 21q22 ITSN1 as a candidate gene in mental retardation. *Leuk Res.* 2010;34:e8–e12. 19679353 10.1016/j.leukres.2009.06.030

[15] Heller PG Glembotsky AC Gandhi MJ Low Mpl receptor expression in a pedigree with familial platelet disorder with predisposition to acute myelogenous leukemia and a novel AML1 mutation. *Blood.* 2005;105:4664–4670. 15741216 10.1182/blood-2005-01-0050

